# Hereditary Gastric and Breast Cancer Syndromes Related to CDH1 Germline Mutation: A Multidisciplinary Clinical Review

**DOI:** 10.3390/cancers12061598

**Published:** 2020-06-17

**Authors:** Giovanni Corso, Giacomo Montagna, Joana Figueiredo, Carlo La Vecchia, Uberto Fumagalli Romario, Maria Sofia Fernandes, Susana Seixas, Franco Roviello, Cristina Trovato, Elena Guerini-Rocco, Nicola Fusco, Gabriella Pravettoni, Serena Petrocchi, Anna Rotili, Giulia Massari, Francesca Magnoni, Francesca De Lorenzi, Manuela Bottoni, Viviana Galimberti, João Miguel Sanches, Mariarosaria Calvello, Raquel Seruca, Bernardo Bonanni

**Affiliations:** 1 Division of Breast Surgery, European Institute of Oncology, Istituto di Ricovero e Cura a Carattere Scientifico (IRCCS), 20141 Milan, Italy; giulia.massari@ieo.it (G.M.); francesca.magnoni@ieo.it (F.M.); viviana.galimberti@ieo.it (V.G.); 2 Department of Oncology and Hemato-Oncology, University of Milan, 20122 Milan, Italy; elena.guerinirocco@ieo.it (E.G.-R.); nicola.fusco@ieo.it (N.F.); gabriella.pravettoni@ieo.it (G.P.); 3 Breast Service, Memorial Sloan Kettering Cancer Center, New York, NY 10065, USA; montagng@mskcc.org; 4 i3S—Instituto de Investigação e Inovação em Saúde, University of Porto, 4200-135 Porto, Portugal; jfigueiredo@ipatimup.pt (J.F.); sfernandes@ipatimup.pt (M.S.F.); sseixas@ipatimup.pt (S.S.); rseruca@ipatimup.pt (R.S.); 5 Institute of Molecular Pathology and Immunology of the University of Porto (IPATIMUP), 4200-135 Porto, Portugal; 6 Department of Clinical Sciences and Community Health, University of Milan, 20133 Milan, Italy; carlo.lavecchia@unimi.it; 7 Department of Digestive Surgery, European Institute of Oncology, Istituto di Ricovero e Cura a Carattere Scientifico (IRCCS), 20141 Milan, Italy; uberto.fumagalliromario@ieo.it; 8 Departments of Medicine, Surgery and Neuroscience, University of Siena, 53100 Siena, Italy; franco.roviello@unisi.it; 9 Division of Endoscopy, European Institute of Oncology, Istituto di Ricovero e Cura a Carattere Scientifico (IRCCS), 20141 Milan, Italy; cristina.trovato@ieo.it; 10 Division of Pathology, European Institute of Oncology, Istituto di Ricovero e Cura a Carattere Scientifico (IRCCS), 20141 Milan, Italy; 11 Applied Research Division for Cognitive and Psychological Science, European Institute of Oncology, Istituto di Ricovero e Cura a Carattere Scientifico (IRCCS), 20141 Milan, Italy; serena.petrocchi@ieo.it; 12 Division of Breast Imaging, European Institute of Oncology, Istituto di Ricovero e Cura a Carattere Scientifico (IRCCS), 20141 Milan, Italy; anna.rotili@ieo.it; 13 Division of Plastic Surgery, European Institute of Oncology, Istituto di Ricovero e Cura a Carattere Scientifico (IRCCS), 20141 Milan, Italy; francesca.delorenzi@ieo.it (F.D.L.); manuela.bottoni@ieo.it (M.B.); 14 Institute for Systems and Robotics, Instituto Superior Técnico, 1049-001 Lisboa, Portugal; jmrs@tecnico.ulisboa.pt; 15 Division of Cancer Prevention and Genetics, European Institute of Oncology, Istituto di Ricovero e Cura a Carattere Scientifico (IRCCS), 20141 Milan, Italy; mariarosaria.calvello@ieo.it (M.C.); bernardo.bonanni@ieo.it (B.B.); 16 Medical Faculty, University of Porto, 4099-002 Porto, Portugal

**Keywords:** gastric cancer, breast cancer, E-cadherin, CDH1 gene, germline mutations, hereditary syndrome, prophylactic surgery

## Abstract

E-cadherin (CDH1 gene) germline mutations are associated with the development of diffuse gastric cancer in the context of the so-called hereditary diffuse gastric syndrome, and with an inherited predisposition of lobular breast carcinoma. In 2019, the international gastric cancer linkage consortium revised the clinical criteria and established guidelines for the genetic screening of CDH1 germline syndromes. Nevertheless, the introduction of multigene panel testing in clinical practice has led to an increased identification of E-cadherin mutations in individuals without a positive family history of gastric or breast cancers. This observation motivated us to review and present a novel multidisciplinary clinical approach (nutritional, surgical, and image screening) for single subjects who present germline CDH1 mutations but do not fulfil the classic clinical criteria, namely those identified as—(1) incidental finding and (2) individuals with lobular breast cancer without family history of gastric cancer (GC).

## 1. Introduction

Since the early discovery of CDH1 germline mutations in Maori kindred [1], clinical interest in this cancer predisposition syndrome has progressively increased. Families affected by CDH1 germline mutations show a strong aggregation for diffuse gastric cancer (DGC) and lobular breast cancer (LBC) [2]. In 1999, the International Gastric Cancer Linkage Consortium (IGCLC), defined specific clinical criteria to select individuals for CDH1 genetic screening and introduced the definition of a new syndrome, the so-called Hereditary Diffuse Gastric Cancer (HDGC) [3]. The detection rate of CDH1 mutations using the first guidelines published by the IGCLC in 1999, was approximately 40% [4]. However, since CDH1 germline mutations were also identified in individuals who did not meet these criteria (such as those with sporadic early onset DGC and those with LBC without a family history of DGC), those criteria for CDH1 genetic testing were subsequently revised [5,6,7]. In individuals who meet the IGCLC 2010 criteria [6], the cumulative incidence of GC at age 80 years is 70% (95% CI, 59–80%) for males and 56% (95% CI, 44–69%) for females. The risk of breast cancer (BC) for females in the same study was 42% (95% CI, 23–68%) [8]. Roberts and colleagues recently reported that in individuals with CDH1 pathogenic variants identified by the MultiGene Panel Testing (MGPT), who did not meet the established clinical testing criteria, the cumulative incidence of GC at age 80 years was significantly lower: 42% (95% CI, 30–56%) for men and 33% (95% CI, 21–43%) for women [9]. The identification of unexpected CDH1 germline mutations in the absence of specific clinical criteria suggests that the HDGC syndrome might be a more complex syndrome than the one originally defined. Additionally, the low frequency of pathogenic variants in countries with high-incidence of GC has opened new discussions about the clinical management of this syndrome. In this multidisciplinary clinical review, the following major topics will be reviewed: (a) GC epidemiology and the strategies to reduce its risk; (b) CDH1 genetic testing in HLBC; (d) clinical and laboratory management of CDH1 missense mutations; and (e) prophylactic surgery, including gastrectomy and mastectomy.

## 2. Hereditary Diffuse Gastric Cancer

**Figure 1:**
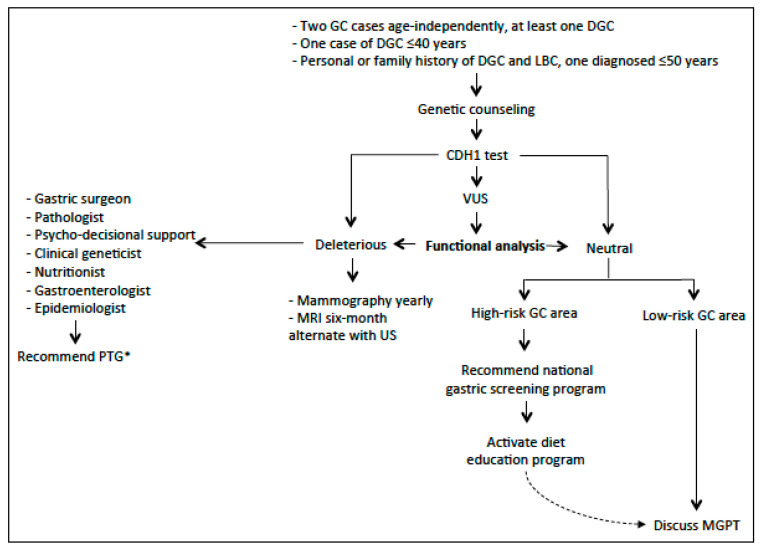
The flow-chart describes the clinical management of hereditary diffuse gastric cancer syndrome [* If refuse PTG (prophylactic total gastrectomy) consider gastric endoscopic surveillance with Cambridge protocol].

In Figure 1, we purposed a flow-chart for the clinical management of the HDGC syndrome.

### 2.1. Environmental Factors and GC

Despite a substantial fall in mortality over the last century, GC remains the third cause of cancer death worldwide [10,11]. Nonetheless, until recently it received little attention both in terms of prevention and research efforts. In 2012, in order to meet this need, a global consortium, the «Stomach cancer Pooling» (StoP) project was launched [12]. The StoP project is a consortium of epidemiological studies including case-control studies, and nested case-controls within cohort studies. The main aim of the StoP project is to examine the role of several lifestyle and genetic determinants in the etiology of GC, through pooled analyses of individual-level data. Helicobacter pylori (Hp) is known to be the major risk factor for non-cardia stomach cancer. An independent effect of sex on the prevalence of Hp infection has been identified—compared to women, men have a significantly higher risk [odds ratio (OR), 1.33, 95% confidence interval (CI), 1.04–1.70]. Cigarette smoking and heavy alcohol consumption are other well-known risk factors for GC, with a 40% excess risk in smokers versus non-smokers [13] and a 50% increased risk for heavy drinkers compared to never drinkers [14]. As for many other cancers, low socioeconomic status has also been identified as a risk factor for GC; in the latest StoP analysis, the pooled OR for the highest compared to the lowest level of education was 0.60 (95% CI, 0.44–0.84) [15]. With reference to dietary factors, data from the StoP project confirmed that meat consumption is a risk factor [16], whereas fruit intake is protective [17]. The interaction of all these environmental factors and HDGC, remains unquantified nowadays.

### 2.2. Pathology of HDGC

The morphology of HDGC encompasses a spectrum of histopathological lesions that should be searched for and characterized in biopsy specimens from CDH1 carriers. The specific lesions in HDGC are tiny foci of typical signet ring cells (SRC), usually confined to the superficial lamina propria, without infiltration beneath the muscularis mucosae. Neoplastic cells are usually small in the deep level at the neck gland zone and enlarge towards the surface. Furthermore, two pre-invasive (or precursor) lesions (pTis) of signet ring cell carcinoma (SRCC) have been recognized exclusively in CDH1 carriers, so far: (1) in situ SRCC, corresponding to the presence of SRC with hyperchromatic and depolarized nuclei within the basal membrane of a gland replacing the normal cells of the gland; (2) pagetoid spread of a row of SRC below the preserved epithelium of glands and foveolae, and also within the basal membrane [18,19].

Endoscopic biopsies specimens from CDH1 carriers might also disclose features of poorly cohesive (diffuse) GC with an “aggressive” phenotype, represented by pleomorphic, bizarre, and diffusely infiltrative cells. These features are highly suggestive of advanced disease. Their presence, along with the coexistence of typical SRC, should be described in the pathology report, to prompt staging and clinical intervention [20].

Criteria for the identification of SRC lesions should be strictly followed in order to diminish the risk of over-diagnosing of mimickers of SRCC or precursor lesions. In the gastrointestinal tract, various benign “signet cell like changes” might mimic SRCC [21,22]. Therefore, confirmation of focal SRC lesions in the stomach by a histopathologist with experience in this area is strongly recommended.

### 2.3. Histopathology of Prophylactic Gastrectomy

Macroscopic examination and sampling of prophylactic gastrectomies (PTG) should follow specific protocols, and the histological examination should be made using a checklist [6].

Pathology data from over 170 total PTG in the setting of HDGC has been published until now [23]. Gross examination revealed HDGC lesions in only a minority of cases (11.7%), encompassing pale patches, nodules, and tiny ulcers/scars. The majority of total gastrectomies from CDH1 carriers exhibit tiny mucosal foci of SRCC or in situ SRCC, although sometimes these were only discovered after careful review by an expert pathologist [18,24,25,26,27,28,29,30,31,32,33].

A recent review [23] demonstrated that when a thorough histopathological examination of the entire gastric mucosa was not performed, HDGC lesions were found in only 62.5% of the total gastrectomies. In contrast, when the whole gastric mucosa was examined according to total-embedding protocol, precursor lesions or invasive carcinoma foci were identified in almost all gastrectomy specimens (95.3%). Moreover, the application of total-embedding protocol considerably increased the number of HDGC lesions identified. These findings argue in favor of the use of the total-embedding protocol and the thorough histopathological examination of the entire gastric mucosa, as the gold standard practice for the evaluation of total gastrectomy specimens from CDH1 carriers. However, in laboratories under resource constraint, it might be impossible to perform total embedding of the stomach. In these cases, the pathologists should clearly state, in the histological report, the percentage of gastric mucosa that was examined.

According to the data published in the current literature, the number of pT1a carcinoma foci found in total gastrectomy specimens from CDH1 carriers, ranged from 1 to 487 and the size varied from <0.1 mm to 16 mm. The number of cancer foci is significantly higher in specimens with previous positive endoscopic biopsies [23]. No correlation between the number, diameter, or location of the HDGC lesions and the age, gender, or CDH1 germline variant of the CDH1 carriers has been described so far. The detection of in situ carcinoma lesions is not as frequent as the detection of pT1a carcinoma foci, suggesting that invasion of the lamina propria by SRC might occur without a morphologically detectable pre-invasive lesion [18,34]. To date, discordant results are published regarding the anatomical location of the cancer foci. Several authors reported a proximal clustering [25,30,31,35] while others described the cancer foci, as dispersed throughout the stomach mucosa [18,20,29]. These findings are in contrast with early reports in New Zealand Maori kindred, where most foci were found within the body-antral transitional zone and in the distal stomach [26,27]. The cause of this variation remains to be clarified. It is essential that the locations of biopsies within gastrectomy specimens are specifically reported, to learn more about the distribution of early HDGC in the stomach.

Finding invasive carcinoma beyond the lamina propria in total gastrectomy specimens from asymptomatic CDH1 carriers is rare [31,33,36,37,38]. Lymph node metastases have been described in one case with invasion of the subserosa (pT3) [36].

Surgical margin status must confirm that there is no residual gastric mucosa and tumor at the margins. However, esophageal cardiac-type glands are scattered in the lamina propria through all levels of the esophagus. The risk to develop SRCC in these glands is unknown but has not been reported [39]. Metaplastic and heterotopic gastric mucosa can be seen elsewhere in the gastrointestinal tract, and a mucosal SRCC was described in the duodenum [40].

Background changes in the gastric mucosa of PTG specimens encompass mild chronic gastritis, which is a frequent finding (49%), sometimes displaying the features of lymphocytic gastritis. Foveolar hyperplasia and tufting of surface epithelium, focally with globoid change, is also a frequent finding and, in some areas, vacuolization of surface epithelium is very striking, however this does not seem to be a specific finding. Intestinal metaplasia and Helicobacter pylori infection were found in 22% and 23% of total gastrectomy specimens, respectively [23].

### 2.4. Histopathology: Advanced HDGC

Advanced HDGC predominantly presents as linitis plastica with diffuse infiltration of the gastric wall. Histology can show predominantly or exclusively SRC (“signet ring cell type” poorly cohesive carcinoma). However, more often these tumors are composed of a pleomorphic neoplastic infiltrate with a small subset of SRC (“non-signet ring cell type” or “not otherwise specified” poorly cohesive carcinoma). In a minority of cases, tumor cells are arranged in small aggregates, sometimes rosettes or glandular-like structures. A component of extracellular mucin might also be present, in which the neoplastic cells float. Although morphological features are not specific, in situ lesions, including pagetoid spread of SRC, in the surrounding non-neoplastic mucosa are important clues for the hereditary nature of the tumor.

### 2.5. Histochemical and Immunohistochemical Stains

The use of histochemical stains for neutral mucins, such as PAS-D is useful for the detection or confirmation of tiny intramucosal carcinomas in which the neoplastic cells are dispersed among preserved foveolae and glands. This should be performed routinely in the examination of gastric biopsies taken during endoscopy and in total gastrectomies from HDGC patients [41]. A cytokeratin stain can help to confirm the epithelial nature of the SRC, if there is any doubt, as well as to confirm the depth of penetration within the gastric wall.

**Figure 2:**
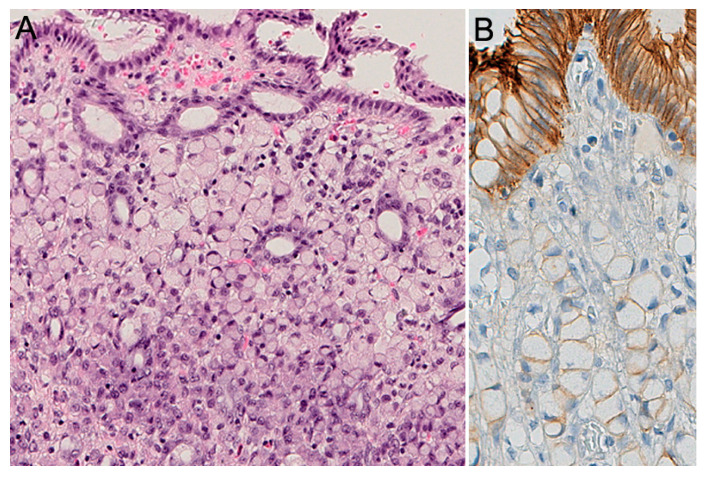
Intramucosal HDGC, pT1a. (**A**) Signet ring cells are larger at the surface, foveolar type; in the deeper zone, at the level of the isthmus, the neoplastic cells are much smaller, immature type. (**B**) E-cadherin expression (IHC) shows absence or marked decrease of E-cadherin at the cell membrane of signet ring cells.

Aberrant immunoreactivity of E-cadherin has been described in precursor lesions, as well as in early and advanced carcinomas. In pagetoid spread of SRC and in situ SRCC, E-cadherin immunoexpression can also be reduced or absent [18] (Figure 2). Heterogenous E-cadherin staining patterns have been described in invasive lesions from CDH1 carriers, including complete loss of expression, reduced membranous immunoreactivity, and “dotted” or cytoplasmic staining [42], however, the pathologist should be aware that E-cadherin expression, as detected by immunohistochemistry, is not always reduced or absent, and can be maintained independent of the presence of CDH1 mutation. Therefore, E-cadherin staining should not be used as a pre-screening method to select patients eligible for germline CDH1 alteration analysis.

Several studies compared the immunohistochemical profile of HDGC with the progression of the disease. According to recent studies, the aberrant expression of p53, increased proliferation activity (evaluated by Ki-67) [42], and over-expression of p16 [43] are emerging biomarkers of progression from indolent to widely invasive HDGC lesions and adverse prognosis.

E-cadherin-null cells have numerous adaptations that affect the cortical actin cytoskeleton. These changes appear to undermine the efficiency of the plasma membrane deformation processes, establishing numerous druggable vulnerabilities [44], yet to be explored for the chemoprevention of DGC and LBC [45].

### 2.6. Endoscopy

For patients with a pathogenic germline CDH1 variant, endoscopic surveillance is the only alternative to PTG (if patient refuses). However, endoscopic detection of SRCC in CDH1 carriers is poor, and histological evaluation of surgical specimens demonstrates cancer foci in up to 45–60% of cases with a negative endoscopic evaluation [46,47]. The main factor that hinders the endoscopic diagnosis of early DGC is that tumor cells begin infiltrating the mucosa, while preserving a normal surface epithelium. Moreover, SRCC foci can be sparse (less than 2% of the gastric mucosa) and each focus is very often less than 1 mm in greatest diameter [29].

For this reason, gastrectomy is advised, regardless of the endoscopic findings [6]. For individuals who refuse surgery, despite carrying a pathogenic variant, a variant of uncertain significance (VUS), or fulfilling the HDGC criteria without having a germline CDH1 mutation, annual surveillance starting at age 20, following the Cambridge protocol, in experienced centers, is recommended [38].

According to the IGCLC endoscopy surveillance protocol, a careful examination in a dedicated session of at least 30 min with high definition white light is recommended. Extensive washing of the mucosa with the assistance of mucolytic and anti-foaming agents is advised, in order to allow for careful evaluation of the entire gastric mucosa. Since the lack of distensibility is a sign of an infiltrative process such as linitis plastica, repeated insufflation and deflation to maximize visualization of the entire gastric mucosa, and a check for distensibility is suggested.

Prior to obtaining random gastric biopsies, targeted biopsies of all suspicious lesions, in particular pale areas (considered more likely to have abnormal SRC), erythema, erosion, or other gastric abnormalities should be taken. After sampling of all visible lesions, five random biopsies should then be taken from each of the 6 anatomic regions—prepyloric, antrum, transitional zone, body, fundus, and cardia, with these groups of biopsies each being sent separately for pathological analysis [6]. Given the large number of biopsies performed, it is recommended to stop anticoagulation, if possible, prior to the procedure.

A model developed by Fujita et al. estimated that for a 90% detection rate, the theoretical number of biopsies necessary is 1768 per patient, but this is not clinically feasible [31]. The main disadvantage of taking an extensive number of biopsies is the formation of scar tissue, which can then mimic the superficial pale aspect of SRCC lesions. Mi et al. showed that targeted biopsies (of typical pale lesions) can result in detection of SRCC foci in more than 40% of patients, this approach has a sensitivity of 28% [48]. However, we have to consider other studies demonstrating that the pale areas are very non-specific for SRC [35,38,49,50]. Further development of endoscopic techniques, such as electronic enhanced imaging techniques, confocal endomicroscopy, magnification and artificial intelligence, is warranted to improve the detection rate of SRCC foci. This procedure is indicated for microscopic visualization of the mucosa during endoscopy at an approximately 1000-fold magnification, and might limit the sampling error of untargeted biopsies [51].

### 2.7. Prophylactic Gastrectomy

The latest IGCLC guidelines recommend PTG, regardless of endoscopic findings, in early adulthood (20–30 years) [6]. New guidelines are expected to be published very soon. PTG remains the recommended option for GC risk management in pathogenic CDH1 variant carriers. However, there is increasing confidence that endoscopic surveillance in expert centers can be safely offered to patients who wish to postpone surgery or to those whose risk is not well-defined.

Family phenotype, especially the proband’s age at diagnosis, should be also taken into account. Statistical models considering quality-adjusted life-years (QALYs) and cancer mortality have been developed. The QALYs is a generic measure of disease burden, including both the quality and the quantity of life lived. PTG in men at 39 years of age results in 32 incremental QALYs, and a lifetime cancer mortality of 8.5%. Whereas for women, the optimal age for PTG is 30 years, with 33 incremental QALYs, and a lifetime cancer mortality of 1.6% [52]. This model is hypothetic.

However, because PTG has a great impact on the quality of life with both physical and psychological downsides, many factors need to be taken into account. First, the cumulative incidence of GC in unselected CDH1 pathogenic variant carrier families, is significantly lower than in families pre-selected for the HDGC criteria [9]. Second, age at diagnosis in the former group is higher and a substantial proportion of families only present with BC [53]. As a consequence, clinical criteria for CDH1 testing should be extended to families with BC cases only and delayed PTG should be considered in selected cases. Another group of particular interest are individuals with a CDH1 pathogenic mutation, without a family history of GC. The clinical management of these patients remain a matter of debate [54].

PTG can be performed either laparoscopically or open, based on the experience of the surgeon. Intraoperative frozen section of the resection margins is recommended to ensure that no gastric mucosa has been left behind [40]. Additionally a D1+ lymph node dissection is usually recommended. Regarding the reconstruction technique—a jejunal pouch reconstruction has been suggested by some surgeons [55] but there are no clear data indicating advantages of this more complex technique over a standard direct Roux-en-Y, which is generally preferred.

The most significant side effect of a PTG results from a potential leak at the esophago–jejunal anastomosis. Pooled data coming from 14 controlled randomized trials of gastric reconstruction, after total gastrectomy demonstrated a mortality rate ranging from 0 to 22% [56], even though the majority of current investigations from high-volume centers report mortality figures less than 3%.

The procedure is also associated with several side effects, such as early and late dumping syndrome, malabsorption, and postprandial fullness. This procedure in fact is associated with the decrease of vitamin B12 and protein absorption, bacterial overgrowth due to loss of parietal and chief cells of the stomach, reflux, dumping, and weight loss. This nadirs after 3–6 months and averages at about 20% of pre-operative weight [57]. Postoperative follow up with experienced dieticians is crucial since postoperative weight loss happens in all patients.

## 3. Hereditary Lobular Breast Cancer (HLBC)

**Figure 3:**
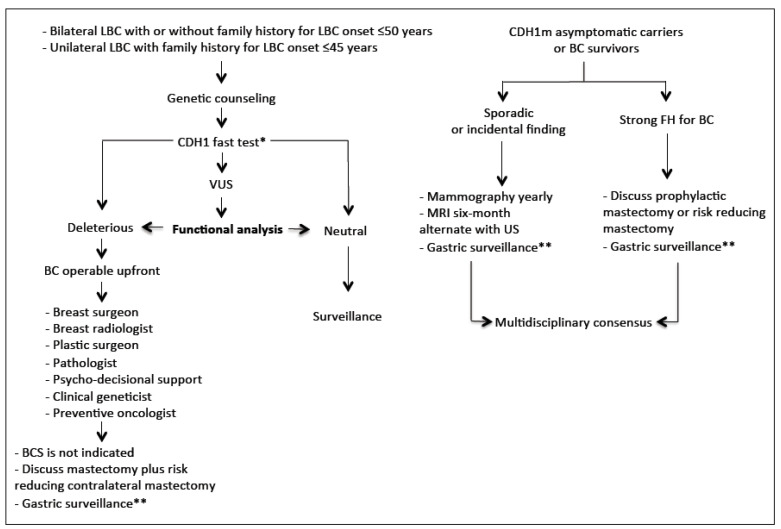
The flow-chart describes the clinical management of hereditary lobular breast cancer predisposition (* In case of CDH1 neg test BRCA1/2; ** Cambridge protocol).

In Figure 3, we purposed a flow-chart for the clinical management of the HLBC syndrome.

### 3.1. Definition

The identification of CDH1 germline mutations in cases of LBC not associated with the classical HDGC syndrome has led to the formation of a working group aimed at better characterizing the genetic susceptibility, the pathophysiology and clinical criteria for this new syndrome, so called HLBC. The working group agreed that the latest clinical criteria for the CDH1 genetic testing proposed by the IGCLC are insufficient to identify patients at risk of HLBC. Therefore, novel criteria have been proposed—(A) bilateral LBC with or without family history of LBC, with age at onset < 50 years; and (B) unilateral LBC with family history of LBC, with age at onset < 45 years. Following the above-mentioned clinical criteria, a CDH1 germline mutation could be identified in 3% of the screened population [7].

### 3.2. CDH1 Screening: Preliminary Considerations

The ongoing trial “Understanding how CDH1 germline mutations affect HLBC” [58] is a clinical genetic study that aims to identify the role of CDH1 in HLBC without DGC aggregation. The first aim of this study is to investigate the prevalence of CDH1 mutations in women with early onset (<45 or <50) invasive or in situ LBC, bilateral LBC, and LBC with no family history of HDGC. To date, 120 patients were enrolled and 6 CDH1 germline variants were identified—1 splice site variant of unknown significance (VUS) and 5 missense (3 VUS and 2 pathogenic) variants. CDH1 germline VUS are under evaluation at the IPATIMUP laboratory in Porto, Portugal to assess their pathogenicity.

### 3.3. Pathology

No specific diagnostic features have been observed in HLBC, compared to non-HLBC. However, appropriate pathological management is crucial to improve patients’ outcome, both in the screening and surgical setting [59]. Akin to LBC, these syndromic tumors are morphologically characterized by the presence of non-cohesive small, uniform, round neoplastic cells that are loosely dispersed throughout a variably dense fibrous stroma or arranged in a linear growth pattern [60]. Most commonly, these tumors are of low or intermediate histological grade (Nottingham grade 1/2) and display a complete or partial loss of E-Cadherin expression [7]. A variable spectrum of non-invasive lobular neoplastic lesions, such as atypical lobular hyperplasia (ALH) and lobular carcinoma in situ (LCIS), are often observed in association with LBC [61]. These non-obligate precursors of invasive BC are also characteristically E-cadherin negative, confirming that alterations in CDH1 are very early oncogenic events in these patients [62]. Given that atypical lobular hyperplasia (AL) and lobular carcinoma in situ (LCIS) are considered risk indicators for subsequent development of BC, we recommend an extensive sampling of the surgical specimen in CDH1 mutations carriers. Likewise, the observation of these non-invasive lobular lesions in bioptic samples in the presence of a possible hereditary gastric and BC syndromes, warrants a multidisciplinary discussion for clinical decision-making [63]. In patients with a history of DGC or LBC, or with documented CDH1 germline mutations, the tumor origin differential diagnosis is not trivial in metastatic settings. Hence, the morphological similarities between these two tumor types can be misleading in the metastatic site. In this setting, despite being nonspecific, cytokeratins 7 and 20 can be helpful, together with GATA Binding Protein 3 (GATA3) and the homeobox protein CDX2 (Table 1). It should be noted, however, that the observation of a metastatic poorly cohesive adenocarcinoma from an unknown primary site remains a clinical problem that requires a multidisciplinary diagnostic management.

### 3.4. Breast Imaging

Due to its non-cohesive histological growth pattern, often without a significant desmoplastic reaction, the detection of invasive LBC on mammography is notoriously difficult [64]. The sensitivity of mammography for the detection of all types of invasive breast carcinomas, ranges from 63% to 98% [65,66]. Due in part to the histopathological features of LBC described above, the sensitivity of mammography in detecting LBC is lower, ranging between 57% and 81% [67,68,69]. The most common mammographic sign of LBC is an irregular, speculated mass, followed by architectural distortion and asymmetries, while microcalcifications are uncommon. The mammographic diagnosis of LBC is also limited by the fact that the density of LBC, on mammography, is similar to, or less than that of the normal surrounding glandular tissue—due to the non-cohesive nature of the LBC cells—challenging the mammographic detection. Recognizing that mammography has a limited value in the detection of LBC, US plays an important role in the evaluation of suspicious physical exam findings. The overall sensitivity of US for the detection of ILC is reported to be between 68% and 98%, and increases with the use of higher frequency probes [70]. The most common ultrasound manifestation of LBC is an irregular mass, with hypoechoic and heterogeneous internal echoes, ill-defined or speculated margins, and posterior acoustic shadowing, findings that are seen in 54–61% of cases [71,72]. Breast magnetic resonance imaging (MRI) has an overall sensitivity of 93% for detecting LBC, similar to the detection of BC overall (90%) [73]. In case of non-indication for prophylactic mastectomy (Figure 3), accurate breast imaging follow-up in CDH1 carriers is recommended. Data regarding the best time interval and best modality is lacking and to date, no international guidelines exist. We recommend the use of annual breast MRI followed by mammography and ultrasound at six months interval, similar to the schedule followed for BRCA1/2 carriers.

### 3.5. Surgical Management

When considering surgical management for CDH1 carriers, several considerations must be made. First of all, it is important to distinguish between carriers who are already affected versus those who have not developed BC yet. According to the recent ASCO 2020 guidelines, neither BRCA nor moderate-penetrance gene mutations should preclude breast-conserving therapy (BCT), when this is clinically appropriate [74]. At present, due to lack of data on contralateral BC risk, risk-reducing surgery in affected CDH1 mutation carriers is not recommended [7]. Similarly, data for recommending prophylactic surgery is lacking. However, mutation status is not the only factor to take into account when considering local therapy decisions; family history, ability to undergo high-risk screening procedures, and patient preference are important factors to evaluate and take into consideration [75]. In our opinion, information on risk-reducing surgery should be provided to carriers with a diagnosis of LBC who have a clinical indication for mastectomy or already had a mastectomy as part of their cancer treatment. Similarly, information on prophylactic surgery should be provided to individuals with a positive family history for LBC and a well-documented CDH1 pathogenic alteration in a first-degree relative [76].

The goal of prophylactic mastectomy is to achieve maximum risk reduction, by completely removing the breast gland but at the same time achieving the best cosmetic result. These aims can be reached through nipple-sparing mastectomy (NSM) with immediate reconstruction, which allows to preserve the skin envelope as well as the nipple-areola complex (NAC). Patients should be informed of the possible surgical and oncological risks of this procedure, which are the risk of local cancer recurrence and the risk of skin necrosis. In terms of oncological safety, the presence of residual breast tissue has raised concerns about this procedure [77]. However, multiple NSM series have been published, and they almost all showed acceptable local recurrence rates [75,78,79]. NSM is also associated with surgical morbidity [75,80], however, in a large series of nearly 2000 consecutive cases from our institution, the rate of nipple necrosis in invasive and in situ cancers was 3.5% and 2.2%, respectively [81], confirming the surgical safety of this technique. Several studies have evaluated the impact of NSM on quality of life [82,83]. A recent systematic review of 22 studies on patient reported outcomes after NSM, 70% of patients were satisfied with their choice, 65% maintained a positive body image, and 95% had no regrets [84].

NSM is technically challenging, as the surgeon must completely and precisely remove the mammary gland by preserving the skin envelope with its sub-dermal vascularization, the nipple, and the inframammary folds [85]. The upper outer linear skin incision is the most commonly used, but surgical approach can be personalized in relation to several clinical peculiarities—indeed, different skin incisions for NSM have been proposed in the literature (hemi-periareolar, round block, vertical pattern, and wise pattern skin incisions) [86].

Robotic NSM is currently being tested in clinical trials. A recent study confirmed technical accuracy, feasibility, satisfaction, and high tolerance rate of patients. The advantage of this technique, compared to the conventional standard NSM, consists mainly in a smaller extra-mammary skin access with the same technical effectiveness [87]. An ongoing randomized clinical trial comparing open NSM and robotic NSM in the prophylactic setting will provide further data on the long-term oncological outcome of this technique [88].

### 3.6. Post-Mastectomy Breast Reconstruction

Breast reconstruction is tailored to each patient, taking into consideration patient anatomy and comorbidities, cancer staging and oncological rules if present, and even patient desires. It requires a close collaboration between a general and plastic surgeon, if a double team approach is used. However, the rates of reconstruction are largely varying, according to different countries and single institutions [89,90], and among women themselves [91,92].

In case of healthy breasts, a conservative mastectomy is usually performed with “aesthetic” surgical incisions. Nipple and areola complex can be preserved. Surgical incisions might be hidden in natural folds of the healthy breast (i.e., inframammary fold, axillary) or just around the areola, resulting in very natural breasts.

With regard to mastectomies for cancer treatment, tumor location and staging certainly influence the surgical approach and skin incisions. For those tumors superficially located, the skin over the tumor is removed within the mastectomy specimen, influencing the resulting scar.

Regardless of the type of mastectomy, options for reconstructions include both prostheses and autologous tissues [90].

Definitive silicone implants or temporary prostheses are conventionally placed below the pectoralis major muscle. More recently, prepectoral reconstructions allow more natural results and lower morbidity [93,94,95]. On the contrary, other patients prefer using autologous tissues, if applicable, because of the similarity to natural breast, stability of the long-term result and anxiety about using foreign materials. Autologous reconstruction encompasses a broad range of procedures incorporating the patient’s own tissues to recreate the breast mound (pedicled and free flaps, fat grafting).

In case of bilateral risk-reducing surgery, the bilateral reconstructions with implants might be really satisfactory, especially in case of small to medium breasts when nipple and areola are preserved, or if reducing patterns in large breasts are successfully used.

Finally, even more challenging are post mastectomy reconstructions after previous conservative surgery and irradiation. It occurs in those patients with hereditary gastric and breast cancer syndromes who have been treated for primary cancer with conservation and who require risk-reducing mastectomies after genetic testing. In fact, mastectomy and reconstruction in irradiated breasts leads to higher postoperative complications, due to impaired flap vascularity and healing process after radiotherapy, the presence of previous scars, and increased capsular contracture in case of implant use. In irradiated breasts, autologous reconstructions are the methods of choice, or more recently, the use of biological matrices and implants has been advocated to decrease capsular contracture rate.

## 4. Common Managements

### 4.1. Genetic Counseling

A multidisciplinary approach can ensure optimal management of the CDH1 germline mutation carriers. Genetic counseling by clinical geneticists with expertise in the field is a critical component of the risk assessment. To identify individuals with HDGC, the clinical evaluation should include collection and review of personal and family history with detailed three-generation family pedigree and confirmation of DGC or LBC diagnoses. The latest IGCLC consensus guidelines [6] suggest CDH1 germline testing in a proband with one or more of the following features—(a) personal history of GC with one or more relatives with GC, regardless of age, in which there is at least one confirmed DGC; (b) personal history of DGC before 40 years; (c) personal or family history of DGC and LBC, one diagnosed before 50 years; (d) personal history of bilateral LBC or family history of two or more cases of LBC diagnoses before 50 years; (e) personal history of DGC and personal or family history of cleft/lip palate; (f) in situ SRC or pagetoid spread of SRC. Criteria will be updated in the new IGCLC guidelines.

Family history should be considered positive in presence of one or more first- or second-degree relatives. It is to note that the suspicion of HDGC could be underestimated in individuals with unknown or limited family history. Initial testing should be considered in an affected proband and when more than one family member is affected with cancer, consider starting from individual with a confirmed diagnosis of DGC or with youngest age at diagnosis. Unaffected individuals should be tested only when affected family member is not available and informed of possible limitations in interpreting test results. An individual (healthy or affected) with a known familial pathogenic (or likely pathogenic) variant could be offered gene testing for the specific familial variant.

Genetic testing in individuals younger than age 18 years could be considered in families with cases of early-onset [96]. Of note, diagnosis of DGC before 20 years is rare [97]. Gene testing of individuals younger than age 18 years requires counseling for both the parents and the child.

Genetic testing can be performed on DNA extracted from blood or buccal samples, except for patients who have received allogeneic bone marrow transplant or with a recent diagnosis of hematologic malignancy (DNA from a fibroblast culture is a preferable sample in these patients).

CDH1 gene analysis should include search of point mutations and large rearrangements by Sanger sequencing and multiplex ligation-dependent probe amplification (MLPA) or by next generation sequencing (NGS). Point mutations (about 30–50% of all variants) might include small intragenic deletions/insertions and missense, nonsense, and splice site variants [4,98]. Exon or whole-gene deletions/duplications have been detected in 6.5% of individuals with HDGC and without pathogenic variants on sequence analysis [99]. CDH1 germline variants are classified according to the IARC 5-tiered classification system in five classes (Class 5: Pathogenic, Class 4: Likely Pathogenic; Class 3: Variant of Uncertain Significance or VUS; Class 2: Likely Benign; Class 1: Benign) [100]. The ACMG variant classification guidelines published in 2015 [101] have recently been revised for the analysis of germline CDH1 sequence variants [102].

During genetic counseling, the likelihood of a positive test, technical aspects, inheritance pattern, and significance of the possible outcomes of testing, such as positive (i.e., pathogenic or likely pathogenic variant), inconclusive or uncertain (i.e., VUS), or uninformative (i.e., no mutations detected), or true-negative (i.e., absence of the known familial mutation) should be discussed. A discussion on life-time risks of DGC or LBC, and PTG, or options of surveillance, should be provided. The counselee should be informed about potential significance of the test results for the family, and about reproductive options, such as the availability of genetic testing through prenatal and preimplantation genetic diagnosis. Genetic counseling should include discussion on the need of a continuous update of VUS. Testing a family member for a VUS should not be offered in clinical setting but could be considered for research purposes. Patients and families suggestive of HDGC with uninformative results (about 50–70% of cases), might have an undetectable defect in CDH1 gene (i.e., mosaicism, sequence variant in an intron or regulatory region, or others not covered for technical limits) or pathogenic variants in other cancer predisposition genes. Therefore, intensive endoscopic surveillance is recommendable for first-degree relatives of patients meeting criteria for CDH1 germline testing with uninformative results. Nowadays, genetic testing includes two main clinical approaches—single-gene testing or MGPT [103,104,105]. The spread of MGPT has led to the identification of CDH1 germline pathogenic and likely pathogenic variants in individuals without a personal and family history, suggestive of HDGC. This kind of results poses major clinical management challenges [106]. Secondary findings could be explained by the presence of families or CDH1 germline mutations with reduced penetrance, and there is a strong need for specific studies in order to obtain more data and drive specific clinical management guidelines.

### 4.2. Psychological Counseling

CDH1 carriers face several challenges, including the burden of cancer treatment, if already affected by DGC or LBC, and the need to decide upon prophylactic surgery if still unaffected. As a result, individuals might experience depressive symptoms [107,108], general distress [108,109,110], and anxiety (109), which can impact their decision-making abilities [111,112,113]. Psychological support is, therefore, a key component of the multidisciplinary management of these patients [114]. To help manage the psychological and medical burden, improve coping skills and the decision-making process, counseling should include a strong psycho-decisional support component [115,116]. The approach should be personalized by taking into consideration the patient’s personality, age, lifestyle, psychological well-being, social support, and self-efficacy [115,117,118,119]. The final aim is for patients to better communicate with their healthcare providers, in order to determine the most adequate preventative, and therapeutic options [120]. Psychological counselling and decision-making support are important tools through which counsellors and health care providers can strengthen patient autonomy and provide informed self-care aligned to the patient’s own deliberative decisions [121]. Empowered patients [122] express higher satisfaction towards the care received, higher trust in healthcare providers [123], better adherence, and less conflict with healthcare providers [124].

This approach is very recent and should be addressed by HDGC and HLBC investigators in the future.

### 4.3. CDH1 Missense Variants: Challenging Routine Laboratory Tests

The implementation of MGPT has led to a dramatic increase in the identification of missense variants. Determining the clinical relevance of such variants is currently a major goal in genomic medicine [125,126,127].

In HDGC, CDH1 missense mutations occur in 22% of cases [128,129] and a large proportion of variants remain unclassified [102]. We, therefore, recommend that a complementary set of analyses encompassing familial and population data, as well as in silico and in vitro tests should be carried out to determine putative variant pathogenicity [130,131]. The assessment of variant effects cannot be achieved by standalone evidence in a single category nor by population, in silico or in vitro tests, since all approaches have limitations [101].

Genetic parameters such as mutation frequency in healthy control population, co-segregation of mutation with the disease within pedigrees, and mutation recurrence in unrelated families should be considered as a first approach [128,129]. Variant frequency in different ethnic groups can be assessed through genomic databases, which compile data from large sequencing consortiums with a few to several thousand participants, namely the 1000 Genomes Project (http://browser.1000genomes.org), the Trans-Omics for Precision Medicine Program (TOPMed; https://www.nhlbiwgs.org/), or The Genome Aggregation Database (gnomAD; https://gnomad.broadinstitute.org/). Nevertheless, databases have limitations, including low-quality data, lack of details on the origin of studies or absence of information regarding possible associated phenotypes [101]. Moreover, the segregation of a variant within a population at low (<1%) or very low (<0.1%) frequencies per se cannot exclude pathogenicity, especially in the presence of clinical and experimental evidence supportive of variant deleterious effects. As HDGC caused by missense mutations has low penetrance [128,132], other host genetic and environmental factors are expected to play a role in the presentation of clinical phenotypes and disease onset. A comprehensive analysis of the pedigree is, thus, crucial to evaluate variant significance and disease risk in germline carriers. Nevertheless, this can be challenging, given the small size of the families and lack of information from patient relatives [128,129].

In silico predictions are valuable tools to estimate the degree of conservation of mutated aminoacids within species, their impact on splicing and, ultimately, on the protein structure [128]. However, the use of multiple programs is mandatory, as different outputs can be obtained, depending on the underlying algorithm [101]. Predictions are based on the principle that aminoacids conserved across species are functionally relevant and their substitution is likely to affect protein function [128,133]. The limitation of this approach is that the degree of conservation of each aminoacid is considered separately and, as such, possible compensatory effects of neighboring positions are not contemplated [128]. Structural modeling is currently suitable to predict the impact of most missense mutations in E-cadherin native-state stability, by covering the major part of protein, including the prodomain, the extracellular, and the catenin-binding domains. A correlation between variants that induce higher energetic penalties and their in vitro loss of function has been clearly demonstrated [133].

In the last few years, a panel of in vitro assays has been developed specifically to assess CDH1 sequence variants [131,134,135,136,137]. The workflow starts by transfecting cell lines with vectors encoding the variant and the wild-type protein. Subsequently, protein expression level, protein localization, and main E-cadherin functions (cell-cell adhesion and invasion suppression) are evaluated [131,134,135]. The CHO (Chinese Hamster Ovary) cell line is the conventionally accepted model to perform all tests, as this cell line is completely negative for cadherin protein expression and displays in vitro invasive properties. Upon transfection with the wild-type E-cadherin, CHO cells acquire the capacity to form cellular aggregates on soft agar and become non-invasive through artificial extracellular matrices [130,134,135]. The effect of missense variants on E-cadherin expression level is assessed by western blot. Low E-cadherin expression strongly indicates structural destabilization and premature degradation of the protein through mechanisms of Protein Quality Control [133]. Immunostaining and its detailed quantitative analysis is then used to address whether the variant induces abnormal patterns of E-cadherin localization [136]. Occasionally, a band mobility shift can also be observed, suggesting aberrant glycosylation of the protein [138,139]. Contrary to wild-type E-cadherin, which is normally present at the plasma membrane, deleterious variants can be diffusely distributed throughout the cell or abnormally accumulated in cytoplasmic regions/organelles [135,136,140]. Remarkably, some deleterious variants are expressed at the plasma membrane, affecting the stability of the interaction between cadherin molecules (on adjacent cells) and increasing protein turnover rates [135,136,141]. Cell–cell adhesive abilities of variants are evaluated by slow aggregation assays. In this technique, a single-cell suspension is seeded on a semi-solid agar substrate and cells with a competent adhesion complex spontaneously aggregate [142]. It is well-established that cells transfected with the wild-type protein form compact cellular aggregates, while cells with dysfunctional E-cadherin form small cellular aggregates or present an isolated phenotype [134,135,143]. Topological features in cell meshes should also be examined to determine morphological and structural consequences upon adhesion loss. Indeed, it was demonstrated that triangles within cell networks of dysfunctional mutants have bigger areas and edges, when compared with those formed by wild-type cells, which indicates that E-cadherin defective cells are loosely attached and display increased protrusion formation [137].

The invasive suppressive potential and interaction of E-cadherin mutant cells with the surrounding extracellular matrix (ECM) are currently being studied using matrigel invasion chambers. Matrigel is the most frequently used ECM in invasion assays and its major advantage lies on its heterogeneous composition [144]. This matrix contains not only structural proteins, such as collagens, fibronectin, laminin, and proteoglycans, but also a panel of growth factors, mimicking the basement membrane composition in vitro [144,145].

A major limitation of in vitro experiments is the time pressure surrounding genetic counseling. Nonetheless, and despite the fact that these assays are low throughput and technically challenging, their results reflect a broad approach evaluating alterations in protein structure, trafficking, cellular signaling, and function, which would be impossible to predict through in silico analysis [130]. By following this analytical pipeline, it is possible to determine the functional impact of 85% of E-cadherin missense variants. However, in 15% of cases, these assays are not sufficient to assure a confident result, and their functional significance remains inconclusive. In such cases, other approaches including cell migration analysis, assessment of the interplay between E-cadherin and its binding partners (by proximity ligation assays), as well as evaluation of downstream targets activation can be applied [135,141,146,147]. In vivo models are also being developed to study variants that still lack a clear functional classification.

Overall, a comprehensive approach aggregating multiple lines of evidence will be crucial to correlate variable effects of missense alterations with disease penetrance and phenotypes. Moreover, given the pleiotropic nature of CDH1 variants, a careful interpretation of data should take into account the disease context, including diffuse GC, LBC, or congenital malformations, so a proper management can be offered to CDH1 variant carriers.

### 4.4. Bioimaging Strategies to Identify Aberrant E-Cadherin Expression Signatures

For most cancer types, to support the diagnosis and orientation of therapeutic strategies, the analysis of specific proteins is often required, as alteration in expression or localization of proteins is commonly observed. Immunofluorescence is currently performed to identify aberrant patterns of E-cadherin expression, indicative of protein deregulation and functional impairment at the cellular level, while maintaining tissue architecture [131,135,138,148]. In parallel with a proper staining method, the adequate acquisition and quantification of E-cadherin expression is mandatory. Several high-resolution microscope imaging modalities, such as time-lapse, confocal laser scanning microscopy (CLSM) and spinning disk microscopy can be used in the acquisition of immunofluorescence images for subsequent examination. However, image analysis is mostly qualitative and strongly operator-dependent [149,150,151], which is likely to influence the biological and clinical evaluation of the data. Quantitative approaches are, thus, essential and required to be implemented for an accurate data interpretation.

Over the last decade, a number of software systems has emerged that aims at the quantification of specific features in microscopy images. Nevertheless, these tools often measure total fluorochrome intensity in defined areas, neglecting the expression profile of the target protein in distinct subcellular/cellular/tissue compartments and possibly failing to recognize events of protein delocalization [152,153]. Additional limitations include the discrepancy of parameters during image acquisition and quantification among different experiments and the lack of tools to take into account cell heterogeneity, with respect to morphology (size and shape). Therefore, in order to overcome the current limitations and in view of the urgent need to implement automatic quantitative tools, a novel bioimaging strategy was recently developed [136,154]. This new pipeline allows for a detailed characterization of protein expression with regard of its level and distribution in intra and intercellular spaces. More specifically, the developed algorithm is able to map specific protein signatures, in images of single cells or cell populations, in a multistep process encompassing automatic selection of cells, networking of cells upon nuclei segmentation and recognition of their geometric centroids [136,154]. Fluorescence signals detected between the centroids of two contiguous cells (internuclear profiles) or in radial profiles from a single cell/centroid are then evaluated. Notably, internuclear profiles are of particular relevance to the study proteins located at the plasma membrane or in specific cellular organelles. In contrast, radial profiles should be employed to capture signals throughout the cytoplasm of single cells and are very useful to investigate cytoplasmic proteins. Overall, and as expected, the compilation of all profiles generates a complex and highly heterogeneous protein map, due to the morphological variability of the cells. Therefore, to solve this drawback, geometric compensation techniques are applied, allowing the extraction of a representative profile of protein distribution in the whole cell population [154]. Geometric compensation is indeed a common procedure used in several image modalities and consists of the estimation of rigid or non-rigid transformations to bring the objects under alignment, as similar as possible in terms of shape and size [155,156,157,158]. Ultimately, a comprehensive dataset of fluorescence intensities and their respective locations is generated. With this innovative approach, a new window of intervention has emerged, not only to identify protein patterns associated with cancer but also to help disclose their related molecular mechanisms.

The value of this pipeline was demonstrated in different studies addressing the functional impact of E-cadherin variants [136,159]. Immunofluorescence images of cells transfected with wild-type E-cadherin and with a panel of cancer-related variants were subjected to the described bioimaging processing. As verified by Sanches and collaborators, cells expressing E-cadherin dysfunctional variants present a distinct profile from that displayed by the wild-type cells [136]. More specifically, the variants induce a significant decrease in fluorescence intensity at the plasma membrane or aberrant intensity peaks in the cytoplasm. Remarkable differences were also detected between the typical virtual cells produced by the wild-type and variant expressing cells, suggesting abnormal E-cadherin trafficking and protein accumulation in distinct cell compartments [136,159]. Based on these results, bioimaging approaches have been proposed as a complementary method to assess the functional relevance of novel E-cadherin missense variants, found in the context of HDGC [6,132]. Interestingly, the same algorithm proved to be efficient in the quantification and mapping of a panel of molecules, including P- and E-cadherin, tubulin, and a mitochondria dye, which are distributed in distinct cellular compartments [154,160]. This could be of particular importance in diagnostic and research laboratories to unravel protein regulation mechanisms or identify predictive biomarkers of the disease.

In the future, a combined approach of quantitative distribution of biomolecules with morphological parameters such as cell area and cytoskeletal organization, would be of great value to determine molecular pathways involved in disease progression, but also in the analysis of drug-screening strategies, pinpointing compounds that better rescue protein scores and, simultaneously, normal cellular phenotypes. Modern machine-learning and data mining techniques could be explored to develop more sensitive and efficient methods for signal detection and quantitative molecular analysis in tissue samples.

## 5. Others

### 5.1. Clinical Management of CDH1 Carriers without a Family History of GC and LBC

The introduction of MGPT for hereditary cancer susceptibility has caused an increase in the number of CDH1 mutations detected [161]. Although rare, such mutations have been discovered in subjects who do not fulfill the clinical criteria established by the latest IGCLC meeting [6]. The cumulative risk of GC and BC in this group of patients is unknown [92] and no management guidelines exist.

In our opinion, in the absence of a clear family history for DGC or LBC, asymptomatic CDH1 pathogenic mutant carrier, should be monitor closely. However, if after multidisciplinary discussion, PTG is opted to be the best approach, the patient should be thoroughly informed of all side effects and of the lack of long-term outcome data.

### 5.2. High and Low-Risk Geographical Regions for GC: Impact on Clinical Management

The worldwide incidence of gastric carcinoma varies greatly by region, so that some geographical areas are considered high- and other as low-risk. The cause of this variability is multifactorial. Interestingly, clinicopathological features of GC in these two areas are different. Lauren diffuse-mixed histotype, younger age, advanced stage, and worse prognosis are more likely in low-risk GC area [162]; whereas clear environmental factors, such as diet, are more frequent in high-risk areas [163,164]. CDH1 mutation frequency (including missense mutations) is higher in low-risk area for GC compared to high-risk areas [165] where GC seem to be “CDH1 independent”.

As a general rule, in high-risk areas, CDH1 negative individuals with a positive family history for GC, should follow diet recommendations and have gastric endoscopic surveillance whereas in low-risk area, these individuals might benefit from MGPT.

## 6. Conclusions

As the understanding of the pathogenesis of this complex syndrome continue to improve, there is a clear need to constantly revise clinical criteria for CDH1 genetic screening. The identification of CDH1 germline mutations in individuals, who do not fulfill the classic IGCLC clinical criteria, raises new questions on how to manage these patients. HDGC syndrome is likely a much more complex disease than what was initially thought. In this review article, we discussed different aspects that should be included in the clinical management of these patients. PTG remains the only life-saving approach for individuals carrying deleterious germline mutations and fulfilling the HDGC criteria, however, great caution is needed in the absence of family history for GC. Different clinical approaches should be considered in different geographical regions. Prophylactic mastectomy should be discussed in CDH1 carriers with a strong aggregation for LBC, fulfilling the established clinical criteria. In asymptomatic CDH1 carriers who do not fulfill the clinical criteria, surveillance is preferred. Risk reducing surgery should be only considered in patients who need a mastectomy or had a mastectomy in the past.

Given the complexity and the rarity of this syndrome, CDH1 carriers should always be treated in a multidisciplinary fashion and in high-specialized cancer centers.

## Figures and Tables

**Table 1:** Panel of expressed markers in diffuse gastric cancer (DGC) and/or lobular breast cancer (LBC).

Markers	DGC	LBC
Cytokeratin 7	+/-	+
Cytocheratin 20	-/+	-
GATA3	-	+
CDX2	+/-	-
